# Knowledge of undergraduate Indian dental students on research methodology

**DOI:** 10.6026/973206300210048

**Published:** 2025-01-31

**Authors:** Harsh Priyank, Sumit Mohan, Butta Viswanath, Gaurav Kumar, Ravishankar Prasad

**Affiliations:** 1 Department of Conservative, Endodontics & Aesthetic Dentistry, Dental Institute, Rajendra Institute of Medical Sciences, Ranchi, Jharkhand, India

**Keywords:** Biomedical research, curriculum, dental education, evidence-based medicine, undergraduate students

## Abstract

The knowledge and experiences of undergraduate Indian dental students towards research is of interest. A cross-sectional observational study was conducted by using a self-explanatory questionnaire consisting of 12 questions. A total of 182 students participated in the study. Many first, second and third-year dental students did not conduct research. However, those in the third and final years enjoyed research activities. Moreover, about 80% of third and final-year students expressed interest in pursuing further research.

## Background:

Research is a methodical procedure that follows certain set rules to produce new research, information, or inventions in a particular field [1, 2]. Health research is described as the process of combining fresh information with scientific techniques to identify and address health-related issues [3]. Hence, scientific research is essential in the field of dentistry as is clinical and academic up gradation. It is a basic skill and an important facet in the dental education curriculum related to health care [4]. The standards of clinical education are designed to ensure that healthcare professionals acquire a comprehensive understanding of scientific principles and develop essential clinical competencies. These standards focus on the integration of theoretical knowledge with practical skills, allowing students to apply their learning in both simulated environments and real clinical settings. To achieve this, clinical education programs are structured to foster skill development in various psychomotor tasks, which are critical for providing safe and effective patient care [5]. In these settings, experienced supervisors and mentors guide students, offering valuable feedback and ensuring they gain hands-on experience. Furthermore, valid and reliable methods of assessment are essential to accurately evaluate the progress and proficiency of learners. This organized approach ensures that healthcare providers have enough resources for the difficulties of practicing medicine while concurrently improving the overall quality of healthcare through assurance of competence in essential clinical skills [6]. Undergraduate dental education focuses on equipping students with the necessary skills to perform a variety of invasive and irreversible clinical procedures. This training takes place under the close supervision of experienced and qualified dental professionals. The goal is for students to gain competence in performing these procedures on real patients, ensuring they are well-prepared to handle the complexities of dental practice. By working directly with patients, students develop both technical expertise and the confidence needed for their future careers [7]. Therefore, providing set up for basic undergraduate dental Research in the BDS curriculum plays an important role and platform for active learning and critical thinking among undergraduate students. The role of dentists extends far beyond addressing dental health concerns; they play a vital part in enhancing patients' self-esteem, appearance and overall well-being. A dentist's ability to restore or improve a patient's smile can have a profound impact on their confidence and quality of life. In addition to restoring function, dental treatments often focus on aesthetic considerations, which can greatly influence a patient's psychological and social well-being [8]. The scope of dentistry involves a combination of science, art and interpersonal skills aimed at improving both the oral and emotional health of individuals. Moreover, dental procedures often involve considerably invasive interventions, such as tooth extractions, root canals, or dental implants. These procedures are performed in a confined oral cavity; where precision is vital [9]. Dentists have to demonstrate remarkable manual dexterity and extreme attention to details in order to ensure proper execution of complicated procedures. The complex nature of these tasks requires both technical proficiency and an ability to maintain composure under pressure. The requirement for precision in dentistry provides the best possible outcome for patients and minimizes the likelihood of complications, underlining the need of skilled hands and extensive training for dental professionals [10]. Fresh dental graduates starting independent clinical practice have several obstacles. Alongside acquiring professional skills, they are required to exhibit strong communication abilities to help reduce patient stress and anxiety during procedures. Effective communication is essential to building patient trust and making sure their comfort during the procedure [11].

Time management arises as an essential skill, as graduates must adeptly manage multiple patients and responsibilities. Further more, upholding elevated standards of professionalism is vital for establishing a favorable reputation and nurturing good relationships with patients. These problems necessitate that dental graduates quickly adapt to the expectations of independent practice, moving from supervised roles to overseeing all facets of a dental clinic. Their capacity to manage these problems is crucial to establishing successful, enduring careers in dentistry [12]. In developing countries like India, inadequate attention given to research in the dentistry curriculum often results in significant deficiencies in scientific understanding among newly graduated dentists. This restriction hampers their ability to stay up to date on the most recent advances in dental science and practice. Incorporating dental research into the undergraduate curriculum is crucial regardless of the intention of students to follow an academic or clinical path [13]. By research engagement, students can cultivate critical thinking abilities; enhance their knowledge of dental principles, while contributing to the development of the dental profession. Moreover, undertaking research with professional supervision will equip graduates with the knowledge to tackle real-world challenges and maintain expertise in evidence-based dentistry practice [14]. Numerous studies underline the critical role of research in undergraduate education, particularly with regard to clinical practice. Research demonstrates an important impact on students' capacity to utilize their knowledge in real-life situations. Research-engaged undergraduate students typically display superior clinical skills and problem-solving capabilities contrasted to their peers who don't participate in research activities [1]. This is particularly noticeable in dentistry institutions throughout India, where the efficacy of research initiatives, including those conducted by undergraduate students, is greatly influenced by multiple factors. These encompass student's previous understanding, individual opinions and genuine curiosity for the subject area. A key component in research effectiveness is the students' willingness to look into unresolved or undiscovered topics [3- 4]. Through research engagement, students not only develop essential skills but also nurture a mindset that encourages an investigative and solutions-oriented approach to clinical challenges. The capacity to investigate unresolved inquiries or unaddressed matters in the area significantly amplifies their contributions to the clinical environment, underscoring the essential importance of research in undergraduate education [5]. Many studies show that even though undergraduate students have previously held positive views regarding scientific research, a significant number choose to pursue clinical practice instead of a career in health research. As a result, a considerable number of dental practitioners exhibit strong clinical skills yet fall short in research capabilities [15]. Consequently, dental educators have to assess the readiness of undergraduates during their medical education and improve their analytical skills from the beginning. Therefore, it is of interest to assess the understanding and experiences of dental undergraduates concerning research at Rajendra Institute of Medical Sciences, Jharkhand.

## Methods and Materials:

This cross sectional study was conducted among all the dental students from first to final years at Rajendra Institute of medical sciences, Jharkhand. Ethical clearance was obtained by the Institutional Ethical Committee bearing registration.

## Sample size calculation:

Sample size calculation was done using the formula:

Sample Size (n)=Z(1-α/2)^2^ x p x (1-p)/d^2^

Where, Z (1 - α/2) = 1.96 for 95% confidence interval

p = Proportion of the participants (32.6%) experienced in research activities.

d = Precision of the study.

With a precision of 7%, the sample size required for the present study is calculated as 182.

## Selection criteria:

The students involved in this study granted informed consent via Google e-forms for voluntary participation.

## Validation of the tool:

A set of 12 questions was constructed in English, derived from the literature research. To guarantee content validity, experts evaluated the questionnaire and computed Aiken's index to determine the relevance of each question, including only those with a score of ≥0.6 in the final version. The reliability of the questionnaire was assessed using Cronbach's α, which varied from 0.76 to 0.89, with a median of 0.85, signifying good reliability.

## Data collection instrument:

The analysis employed a survey comprising three sections. The initial portion comprised the gathering of essential demographic information, whilst the subsequent section evaluated students' understanding of research at RIMS Ranchi, Jharkhand. The final portion evaluates the sources of knowledge utilized by undergraduates during their studies.

## Study procedure:

A closed-ended, standardized and comprehensible questionnaire was designed and administered to 182 dentistry students to collect adequate data for the study. The survey included 12 questions and was accompanied by a consent form as a supplementary document.

## Results:

The research was conducted on 182 students from different BDS years. The majority of the students who participated in the study were females (80%) and a major proportion of students belonged to the 1st year (30%) (Table 1). Most of the students from the third year (56%) and final year (100%) had done various research works during the undergraduate course. The students from third year (94%) and final year (100%) considered research as an important part of the dental curriculum. The students from the third year (94%) and final year (80%) would like to do further research in the future. Among those who had conducted the research, the majority (43.4%) were 4th year students and only 13% of them had voluntarily taken part in the research (Table 2). Statistical analysis using chi square test and T test was performed and findings are summarized as under (Table 3).

It was concluded that,

[1] There is a significant difference between third-year and final-year students, with final-year students having higher participation.

[2] Consider Research Important: No significant difference, as both groups consider research highly important.

[3] Interest in Future Research: There is a significant difference, with third-year students showing higher interest in future research.

[4] The difference in future research interest between third-year and final-year students is statistically significant.

Majority of the students referred lecturer notes, dental text books and other medical books for study purpose. A significant proportion of students from third year (53.4%) and final year (68.2%) follow clinical guidelines during postings. Disappointingly, a very small fraction of the study participants from 4th year (15.6%) had referred journals during their undergraduate teaching. (Table 4) All the study participants from third and final year would like to do post-graduation in the future. In a study conducted among medical students, the barriers faced for doing research was asked, 53% said that lack of awareness was a study. However, in our study, 40 % of the undergraduate students considered inadequate knowledge about research methodology as a barrier. 25 % of students had lack of time and 12 % of the students in our study said lack of interest as a barrier. 8 % of the participants in our study consider inadequate facilities and funds as a barrier [16] (Table 5).

## Discussion:

The importance of regular research in the dental field has become increasingly evident in recent years. Dental practitioners rely on scientific evidence from research to make educated choices in their practice, ensuring that their approaches are based on the most current and reliable knowledge available. The increasing focus on evidence-based dentistry has brought out the importance for dental educators to prepare undergraduate students with the skills and expertise required for delivering effective patient care in clinical settings [17]. By integrating students in research projects, educators promote an extensive explanation of dental science and develop critical thinking abilities. These skills are necessary for addressing complicated problems faced in practice as well as stay informed about the most recent advances in the profession. In addition, exposure to research allows students to analyze clinical evidence, comprehend its significance and incorporate it into their patient care approaches [18]. This not only boosts their skills but also develops a lifelong learning orientation, which is crucial in a field where techniques, materials and technologies are ever evolving. Research is essential in equipping dental students to become skilled, evidence-based practitioners capable of providing the highest degree of patient care [19]. Research studies have demonstrated that students with a solid foundation in research are far more motivated toward pursuing academic professions, where they can combine their clinical skills with advanced research abilities. This combination enables them to move forward in the dental sector, promoting innovation and upgrades in patient care [20]. Moreover, engaging in research during their studies enhances students' critical thinking skill sets, allowing them to analyze complex issues, analyze facts and make decisions with confidence. These talents are essential for both academic achievement and proficient medical practice, where having the ability to make educated choices is critical [21]. Participation in research encourages cooperation and since students frequently engage in collaborative endeavors, acquiring skills in effective communication, exchange of ideas and pursuit of shared goals. This collaborative environment fosters the development of interpersonal skills crucial in professional settings, where collaborative effort and interaction with patients and coworkers are necessary for delivering high-quality treatment [12]. The study conducted by Abu-Zaid *et al.* found that female second-year undergraduate students generally have a positive attitude toward research activities [18]. In contrast, research by Burgoyne *et al.* revealed that male students tend to feel more confident about engaging in research compared to their female counterparts. Moreover, female students often show less interest in research pursuits and tend to prioritize academic work over research involvement [19]. A study by Moraes *et al.* revealed that while 81.7% of medical students expressed interest in research, only 4.7% of the participants considered research to be important [22]. Similarly, in a study by Giri *et al.* most students acknowledged the importance of research; however, 75% had not been involved in any research-related activities, a notably high percentage [23]. In contrast, our study found that nearly all final-year participants had engaged in research, which is a significant difference compared to the findings in previous studies [2, 5, 24, 25, 26, 27, 28-29]. In a study by Möller *et al.* only 12% of medical students had attended an international conference. In contrast, our study found that 53% of participants had presented their research at a conference, a significantly higher percentage than in the previous study [24]. Our research also indicated that most third- and final-year BDS undergraduate students demonstrated a strong understanding of research ethics and the process of presenting papers at conferences. These findings contrast with other studies, where the study subjects had limited knowledge about research work [30, 31].

Moreover, while several participants in our study valued engaging in research beyond their academic curriculum, a study involving dentistry students in Saudi Arabia by AlSayegh *et al.* revealed that these students demonstrated positive attitude towards research, but had low level of knowledge and participated very infrequently in them [25]. This study has certain limitations that must be acknowledged when evaluating the results. A major limitation is the relatively small sample size of 182 participants, which may affect the generalizability of the results. An increased sample size would probably yield more dependable and statistically significant outcomes, providing a more distinct explanation of the observed trends and patterns. A further disadvantage is the homogeneity of the study population, which mainly comprised students from identical backgrounds and experiences. The absence of diversity in the sample may limit the scope of the findings to other groups. The study concentrated on first - and second-year undergraduate dental students, a significant number of whom possessed minimal research experience. Consequently, the findings may not comprehensively reflect the viewpoints and abilities of students with higher-level academic capabilities. Subsequent study with a bigger, diversified sample, together with participants having varying degrees of research experience, may yield deeper and more accurate perspectives on the topic.

## Conclusion:

There is a growing consciousness among Indian dentistry students concerning the relevance of research in their profession. However, several students faced problems conducting research owing to inadequate information and lack of experience. Therefore, it is essential for dental professors to introduce Indian students with the fundamental concepts of biostatistics and research methodologies early in their academic careers by providing access to essential resources which will enhance their research capabilities and comprehension.

## Figures and Tables

**Table 1:** Details of the students

**Total Students N=222**		
**Students participated in the study n=182**		
BATCH	BOYS' n (%)	GIRLS' n (%)
BDS I YEAR	18 (32.14%)	38 (67.85%)
BDS II YEAR	4 (8.1%)	37 (91.9%)
BDS III YEAR	10 (7.5%)	36 (92.5%)
BDS IV YEAR	4 (34%)	20 (66%)

**Table 2:** Attitude, experiences of undergraduate dental students towards research

**Question**	**First Year**	**Second Year**	**Third Year**	**Final Year**
Did you do any research during your undergraduate course?	30%	38%	56%	100%
Was this research part of your curriculum?	30%	38%	56%	100%
Was the research voluntary?	65%	8%	12%	13%
Did you enjoy the research?	15%	38%	56%	87%
Did you get support and help from your teachers when doing research?	25%	38%	56%	93%
Would you have like to have done more research?	25%	69%	94%	80%
Would you like to do post-graduation?	65%	77%	100%	100%
Do you think research is important?	60%	85%	94%	100%
Do you think you will do research in the future?	85%	69%	83%	97%
Do you think research is important, but it is best left to others?	35%	15%	44%	27%
Have you ever read a research paper or publication in a journal?	75%	69%	87%	93%
Have you ever done a paper presentation in a conference	30%	31%	19%	53%

**Table 3:** Statistical interpretation

**Test**	**Category**	**Third-Year Students**	**Final-Year Students**	**Test Result**	**Conclusion**
Chi-Square Test	Research Participation	56 (56%)	100 (100%)	Chi-Square = 56.42, Critical Value = 3.841	Significant difference, reject the null hypothesis
Chi-Square Test	Consider Research Important	94 (94%)	100 (100%)	Chi-Square = 1.26, Critical Value = 3.841	No significant difference, fail to reject the null hypothesis
Chi-Square Test	Interest in Future Research	94 (94%)	80 (80%)	Chi-Square = 5.07, Critical Value = 3.841	Significant difference, reject the null hypothesis
T-Test	Interest in Future Research (Mean)	Mean = 4.7, SD = 0.5	Mean = 4.2, SD = 0.8	T = 5.29, Critical Value = 1.96	Significant difference, reject the null hypothesis

**Table 4:** Daily or weekly use of information sources as an undergraduate

**Source of Information**	**First Year**	**Second Year**	**Third Year**	**Final Year**
Colleagues	81.20%	75.80%	97.90%	93.80%
Lectures/Notes	91.30%	80.60%	53.20%	56.30%
Dental Text Books	92.80%	97.50%	87.50%	97.60%
Other Text Books	97.80%	95.40%	91.30%	99.10%
Clinical Guidelines	17%	21%	53.40%	68.20%
Journals	0%	4.50%	3.50%	15.60%

**Table 5:** Barriers faced by undergraduate students in doing research work

**S.no**	**Barrier to doing research work by the participants**	**Percentage (%)**
1.	Inadequate knowledge of research methodology	40
2.	Time restraint	25
3.	Lack of interest in research work	12
4.	Lack of motivation	8
5.	Inadequate facilities and funds in the college	8
6.	Lack of guidance from college staff	7
